# Fractures of the trochanteric region in children and young adolescents—a treatment algorithm for a rare injury

**DOI:** 10.1007/s00431-020-03816-z

**Published:** 2020-10-24

**Authors:** Stephan Payr, Ellen Payr, Britta Chocholka, Manuela Jaindl, Monika Luxl, Elisabeth Schwendenwein, Thomas Tiefenboeck

**Affiliations:** Department of Orthopedics and Trauma Surgery, Division of Trauma Surgery, Medical University of Vienna, Waehringerguertel 18-20, 1090 Vienna, Austria

**Keywords:** Trochanteric fractures, Children, Adolescence, Treatment algorithm

## Abstract

For femoral fractures of the trochanteric region in children and adolescents, only two mechanisms have been identified to cause a fracture of the proximal femur: high-energy trauma or predisposing bone pathologies with inadequate trauma (e.g., simple fall, movement). We identified 20 patients between 1993 and 2018 with a trochanteric fracture under the age of 18 (12 males; 8 females; mean age, 12 years; range, 4–17 years) who were treated operatively at our department. The mean follow-up of all patients was 50.06 months. All 20 patients were treated operatively. Complications occurred after a mean time of 6.27 months (range, 0.47 to 12.07 months) in two patients. Harris Hip Score was evaluated in all patients with a mean score of 94.16 (range 11 to 100). Eighty-five percent of the patients reached an excellent clinical outcome after treatment. *Trochanteric femoral fractures in children and adolescents are very rare accounting for only 1% of all trochanteric fractures. Excellent long-term results can be achieved with an adequate fracture reduction.*

*Conclusion*: Physicians treating pediatric trauma have to be aware of other predisponding diseases when low-energy trauma leads to a trochanteric fracture as in this study, 50% of the trochanteric fractures were associated with bone pathologies.

**Table:** 

**What is Known:** *• Trochanteric femoral fractures in children and adolescents are very rare* *• In all patients with trochanteric femoral fractures, malignancies have to be ruled out* **What is New:** *• Awareness of an underlying bone pathology in a high number of cases* *• Awareness for necessity of a good fracture reduction leading to highly satisfactory results*

**What is Known:**

*• Trochanteric femoral fractures in children and adolescents are very rare*

*• In all patients with trochanteric femoral fractures, malignancies have to be ruled out*

**What is New:**

*• Awareness of an underlying bone pathology in a high number of cases*

*• Awareness for necessity of a good fracture reduction leading to highly satisfactory results*

## Introduction

Femoral fractures of the trochanteric region in the pediatric population are very rare accounting for less than 1% of all fractures in children [1, 2]. Generally, there are only two mechanisms to cause a fracture of the proximal femur in children: high-energy trauma or in case of inadequate trauma, predisposing bone pathologies [3]. If there is a fracture caused by an inadequate trauma (e.g., simple fall, movement), the underlying disease (pathologies in bone metabolism, preexisting bone deformation, various benign/malignant bone tumors) has to be identified and treated [3].

Due to the rareness of these fractures, no evidence-based management is known. Transepiphyseal, transcervical, and displaced cervicotrochanteric fractures, however, generally require closed/open reduction and internal fixation to avoid complications [4, 5]. However, timing of treatment (early vs. delayed) seems a crucial factor for outcome [5]. In toddlers and non-displaced fractures, a conservative approach (e.g., casting to rest and traction) is a therapeutic option [6]. As fractures of the trochanteric region are not affecting the epiphysis, femoral head necrosis or relevant growth disturbance is not expected.

Though, complications such as coxa vara deformity, leg length differences, and non-union are described [2, 7].

In literature, most information regarding these fractures is found in collectives of pediatric hip fractures with the focus set on femoral neck fractures [1, 5, 8–13]. Predisposing factors for poor outcome or fracture complications, such as non-union or femoral head necrosis, are described in literature. This study is dealing with the surgical treatment of these rare fractures of the trochanteric region in children and adolescents and is the most comprehensive case series in recent literature. Furthermore, it was the goal to increase awareness of the different methods of fixation available for these fractures in children and adolescents.

## Material and methods

We identified 20 patients between 1993 and 2018 with trochanteric fractures under the age of 18 who were treated at our department.

Diagnosis was based on recognized radiological and clinical criteria. All patient information, disease, and treatment-related data were retrieved by a review of the patients’ charts. Prior to this investigation, the corresponding institutional Review Board approved this study.

**Fig. 1:**
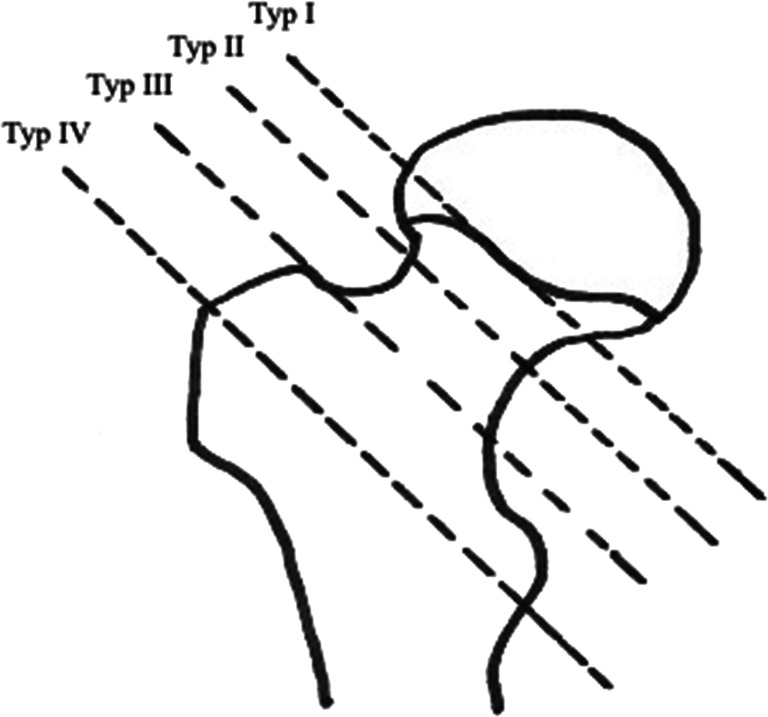
Classification according to Delbet (type I–IV). Type I, transphyseal; type II, transcervical; type III, cervicotrochantric; and type IV, intertrochanteric (pertrochanteric). Accordingly, pertrochanteric fractures are classified as Delbet type IV

**Fig. 2:**
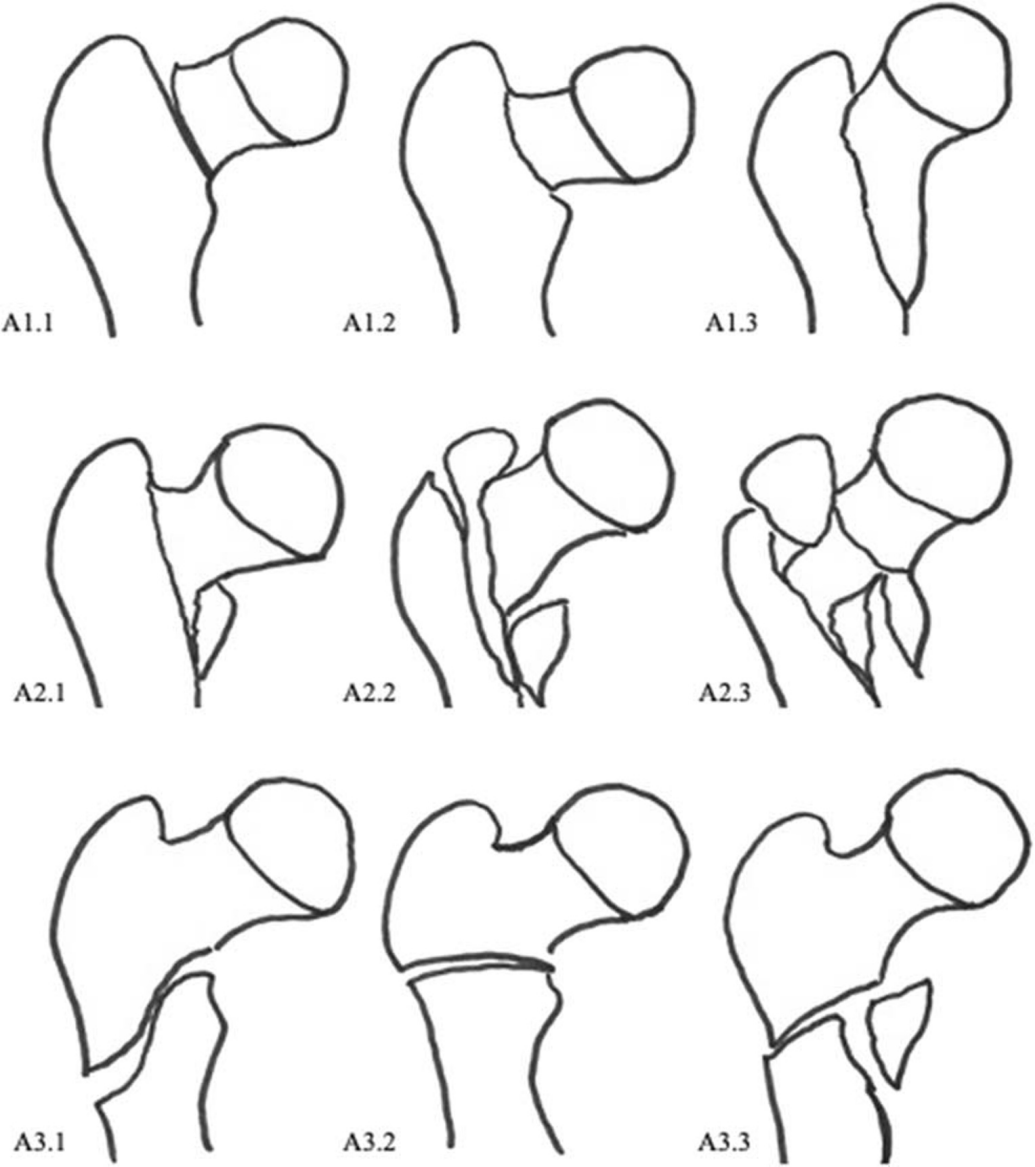
AO classification of pertrochanteric fractures according to Müller et al. A1 Simple (2-part) fracture in the pertrochanteric region. A1.1 Fractures along the trochanteric line. A1.2 Fractures through the greater trochanter. A1.3 Fractures below the minor trochanter. A2 Multifragmentary pertrochanteric fracture. A2.1 With one intermediate fragment (the minor trochanter is detached). A2.2 With two intermediate fragments. A2.3 With more than two intermediate fragments. A3 Intertrochanteric fractures. A3.1 Simple, oblique. A3.2 Simple, transverse. A3.3 With a medial fragment

We analyzed the data of 20 patients with trochanteric fractures (12 males; 8 females; mean age, 12 years; range, 4–17 years). Hip fractures of the growing skeleton are classified after Delbet [14] and Colonna [15] (type I–IV). Accordingly, pertrochanteric fractures are classified as Delbet type IV (Fig. 1). As per- et subtrochanteric are not adequately represented, Arbeitsgemeinschaft Osteosynthese (AO) classification according to Müller et al. (Fig. 2) [16] was added for all fractures included in order to be able to further classify Delbet type IV fractures. In order to radiologically describe the displacement of fractures, displacement was defined in non-displaced, cortical thickness (1–2 mm), half shaft displacement, and total shaft displacement. X-ray images were evaluated independently by 3 trauma surgeons.

Postoperative events such as infection, bleeding, nerve palsy, femoral head necrosis, and pseudarthrosis (non-union) were defined as major complications potentially requiring further surgery.

All patients were followed closely according to the standard follow-up protocol of the Department of Trauma Surgery. The standard follow-up protocol for patients with trochanteric fractures foresees clinical and radiographic examination of the site after 1, 3, 6, 12, and 24 months.

Follow-up protocol includes anterior-posterior and lateral radiographs as well as clinical examination at every follow-up date. Postoperative function and subjective data were assessed by trauma surgeons.

### Statistical analysis

Descriptive data (mean, median, range, proportions) are reported for the entire patient cohort. Statistical analysis focused on surgical and functional outcome after treatment of trochanteric fractures in children and adolescence. Clinical, therapeutic variables (surgery and function), and demographic variables (sex, age, and follow-up) were examined.

## Results

Table 1 illustrates an overview of patient’s characteristics of 20 patients treated for a trochanteric fracture at the Department of Trauma Surgery.

**Table 1:** Patient characteristics, surgical therapy

No. pat.	Age in years	Sex	Fracture type	Trauma mechanism	Time until fracture healing in months	Complications	FUP in months
1	13	M	31 A2.2	Polytrauma	3	No	81
2	11	F	31 A3.3	Fall (< 3 m)	3	No	114
3	13	M	31 A3.3	Fall (epiphysiolysis)	4	No	71
4	10	F	31 A3.1	Polytrauma	3	No	7
5 +	17	M	31 A1.1	Fall (osteosarcoma)	2	No	5
6	17	F	31 A3.2	Fall (osteogenesis imperfecta)	4	No	46
7	15	F	31 A1.3	Fall (bone cyst)	24	Yes	43
8	4	M	31 A3.3	Polytrauma	4	No	7
9	13	M	31 A1.1	Fall (hip dysplasy)	4	Yes	4
10	17	M	31 A3.1	Fall (epiphysiolysis)	14	No	32
11	16	F	31 A2.2	Polytrauma	35	No	101
12	16	M	31 A2.2	Polytrauma	5	Yes	5
13	8	M	31 A1.1	Fall (bone cyst)	2	No	37
14	10	M	31 A3.1	Bicycle	7	No	31
15 -	6	F	31 A3.1	Fall (osteomyelitis)	?	No	
16	16	M	31 A3.1	Fall (> 3 m)	3	No	19
17	17	M	31 A3.2	Polytrauma	3	No	6
18	6	M	31 A1.1	Playing soccer	3	No	13
19	5	F	31 A3.1	Fall	1	No	2
20	4	F	31 A3.2	Fall	1	No	5

*FUP* follow-up

^-^Further treatment in other hospital

^+^Death due to underlying disease

The 20 patients included 12 males (60%) and 8 females (40%) with a median age of 12 years. According to the AO classification, there were four fractures classified as 31 A1.1, one as 31 A1.3, three as 31 A2.2, six as 31 A3.1, three as 31 A3.2, and three 31 A3.3. Eighteen (90%) of these fractures were displaced. Two fractures were not displaced involving an osteosarcoma and a large bone cyst that indicated surgery. The other 18 fractures were displaced (9 half shaft and 9 total shaft displacement). These 18 fractures also showed an axis deviation, 17 in the axial X-ray with a mean angle of 41° (min. 7°; max. 90°), and one in the anterior-posterior view of 35° in varus displacement. In six patients, additional fractures were found (these patients were polytraumatized); in two patients, previous epiphysiolyses were found in medical history; in two patients, juvenile bone cysts were found incidentally; one patient presented with an osteosarcoma, one patient presented with muscular dystrophy, and one with osteogenesis imperfecta. The osteosarcoma patient was treated in cooperation with the department of orthopedics, division of tumor orthopedics. One patient prior to the accident had surgical sanitation of osteomyeolitis, one had a fall from great height (> 3 m), one had a history of fibrous dysplasia, one had a bicycle accident who was treated conservatively abroad, one while playing soccer, and two patients fell from heights (< 3 m).

The leading symptoms were pain in all patients followed by external rotation and limitation of movement along with swelling. All 20 patients underwent surgical treatment. Seventeen patients less than 48 h vs. 3 patients over 48 h until surgery; in detail, one patient was operated on in an outside hospital before attending our department. Three patients were treated within 24 h. Two patients were operated on within a week after the initial trauma, but these patients were initially treated conservatively abroad. One patient was initially treated abroad with a tibial extension for 4 weeks followed by a cast immobilization for another 4 weeks until the patient finally got operated on with an angle plate (patient with a juvenile bone cyst, trauma mechanism fall from a table) to stabilize the fracture at our department. Except for these seven patients, all other 13 patients were operated on immediately, as soon as being empty-stomached (6 h after the last meal or drink), when attending the department.

Surgical treatment consisted of six gamma nails, two dynamic hip screws, and four external fixators; in three patients, the fracture was reconstructed with a plate, in one patient with K-wires, in two patients an angle plate was used, one patient was treated with TEN nails, and one with Prevot nails. In two patients, complications occurred after operative treatment requiring further surgery.

**Fig. 3:**
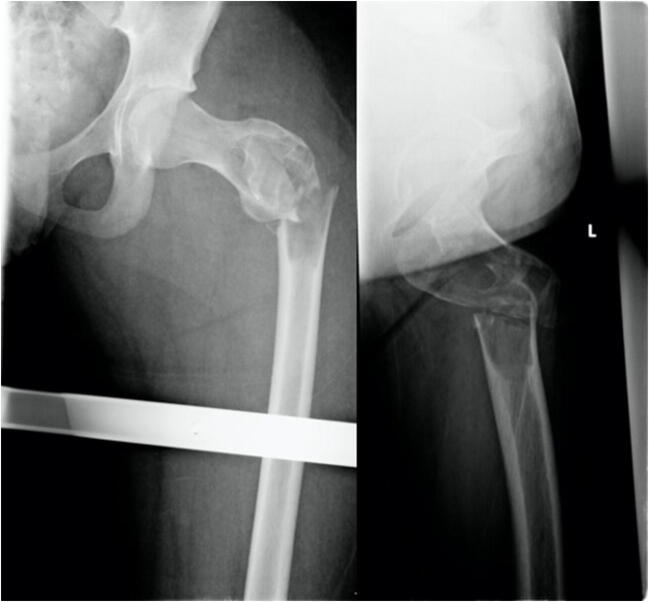
Anterior-posterior and axial X-ray of a displaced trochanteric fracture in a 16-year-old boy with a bone cyst. Full shaft displacement and an axis deviation of 70°

**Fig. 4:**
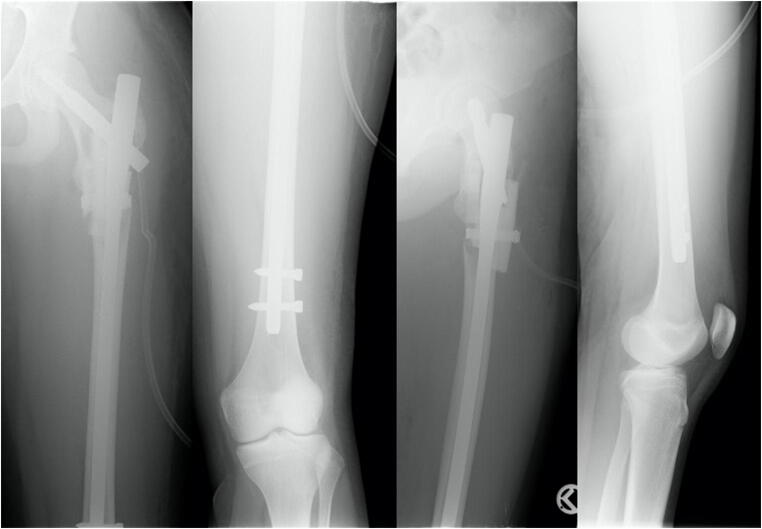
Anterior-posterior and axial X-ray of the left femur after surgical stabilization with a long gamma nail and a cerclage

**Fig. 5:**
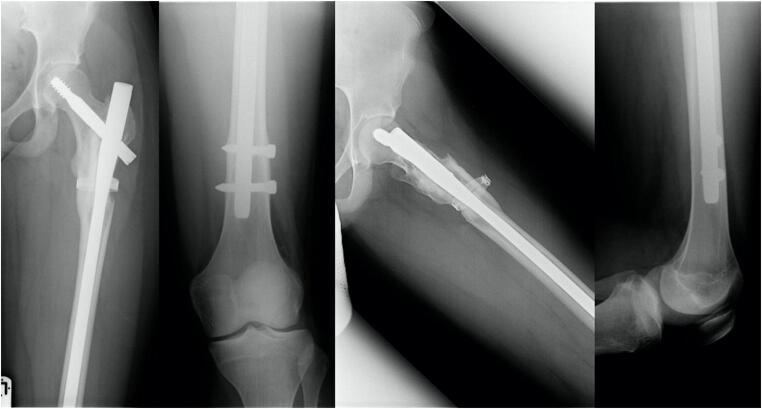
Anterior-posterior and axial X-ray of the left femur showing an atrophic pseudarthosis of the lateral cortex near the implantation site of the femoral neck screw (1 year postoperative)

**Fig. 6:**
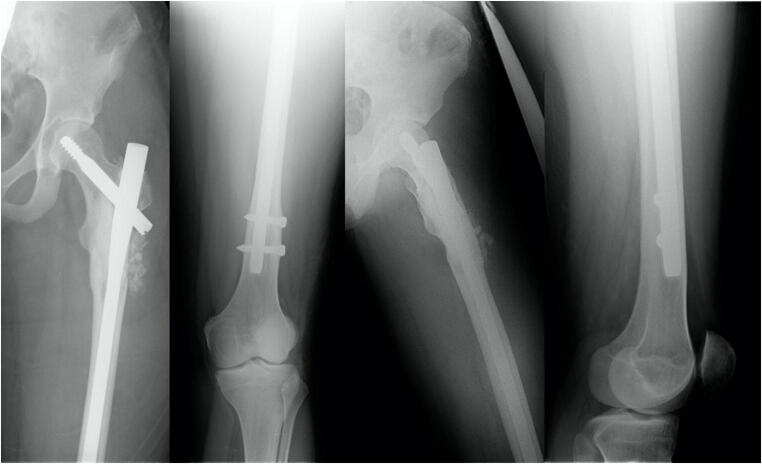
Anterior-posterior and axial X-ray of the left femur after spongioplasty

**Fig. 7:**
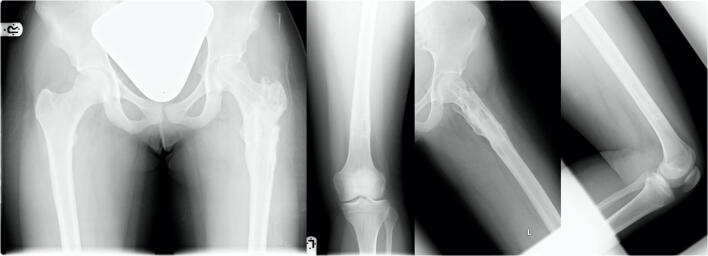
Anterior-posterior view of the pelvis; anterior-posterior and axial X-ray of the left femur healed fracture at the latest follow-up (4 years after injury) after implant removal

Complication 1 occurred in a 15-year-old boy who fell in school and showed a juvenile bone cyst in the major trochanter. Patient got a long gamma nail. Follow-up 1 year postoperatively revealed an atrophic pseudarthrosis of the lateral cortex near the implantation site of the femoral head screw, so it was decided to perform a spongiosaplasty. Last images show a completely healed fracture after implant removal (Figs. 3, 4, 5, 6, and 7).

Complication 2 occurred in a 16-year-old boy involved in a car accident without wearing his safety belt. The pertrochanteric fracture on the left side of this polytraumatized patient was primarily stabilized with an external fixator. During hospitalization, a hematoma compromised the skin (but no signs of compartment) in this region and was incised. Under pressure therapy was necessary for 10 days then wound closure could be performed.

The mean follow-up of all patients was 50.06 months (range, 2.5–250 months). One of the patients was lost for follow-up and underwent further treatment in another hospital. One patient died because of an osteosarcoma as primary disease, 5.7 months after operative treatment at our department.

The mean length of hospital stay in all patients was 19 days (range, 0 to 56 days) including 6 polytraumatized patients with a mean hospital stay of 32 days (min. 14; max. 56 days). The other 14 patients had a mean stay of 10 days (min. 3; max. 25 days).

Harris Hip Score was evaluated in all patients with a mean score of 94.16 (range 11 to 100). Eighty-five percent of the patients reached an excellent outcome after treatment (Table 2 outlines postoperative outcome of all patients).

**Table 2:** Postoperative outcome of all patients

No. pat.	Post-operative ROM	External rotation	HHS*
1	10-0-120	50-0-40	100
2	0-0-110	30-0-50	100
3	10-0-120	50-0-40	100
4	10-0-120	50-0-40	100
5+	10-0-90	20-0-30	89
6	10-0-120	50-0-40	100
7	10-0-100	30-0-50	100
8	0-0-110	30-0-50	100
9	-	-	11**
10	10-0-120	50-0-40	100
11	0-0-110	40-0-30	100
12	0-0-90	20-0-30	89
13	10-0-120	30-0-50	100
14	10-0-120	50-0-40	100
15-	n.e		
16	10-0-120	50-0-40	100
17	10-0-120	50-0-40	100
18	10-0-120	50-0-40	100
19	10-0-120	50-0-40	100
20	10-0-120	50-0-40	100

^*^HHS Harris Hip Score at latest follow-up

^**^Patient with muscle dystrophia

^+^Death due to underlying disease

^-^Transferred to another hospital

Sixteen (80%) out of 20 patients got their implants removed after a mean time of 14.73 months (range, 1.77 to 42.27 months).

## Discussion

Femoral fractures of the trochanteric region in children and adolescents are very rare accounting for less than 1% of all fractures in children [1, 2]. In general, there were only two mechanisms identified to cause fracture of the proximal femur; high-energy trauma or in case of inadequate trauma (e.g. simple fall, movement), predisposing bone pathologies have to be identified [17] which was confirmed in our case series. If a fracture is caused by an inadequate trauma, an underlying bone pathology has to be evaluated.

Fractures of the proximal femur in children often lead to complications in fracture healing caused by avascular necrosis (AVN) of the femoral head or neck, non-union, length discrepancy, osteoarthritis, and an abnormal position of the proximal femur (e.g., coxa vara) [2, 18, 19]. These complications are less frequently observed in extracapsulary trochanteric fractures than in other hip fractures [5, 20–22], as it was also shown in our case series. However, Togrul et al. reported AVN in 2 out of 12 patients suffering from non-displaced Delbet type IV fracture. No other complications were described. The herein reported overall clinically good outcome might also be achieved by early onset of treatment (17 patients less than 48 h vs. 3 patients over 48 h until surgery) and the benign fracture type (Delbet IV) when compared with other hip fractures. We observed complications in only two patients because of one delayed healing and one wound healing disturbance requiring further surgical intervention.

In adults, several classification systems have been used for pertrochanteric and subtrochanteric fractures, mostly common is the AO classification. Although this classification system has not been commonly used in pediatric patients, we used the AO classification by Müller et al. [16] to provide more information about the different patterns and severity of pertrochanteric and per- et subtrochanteric fractures. This would not be possible using only the Delbet [14] classification, which allows no further sub-division of the type IV.

Numerous treatment options have been proposed and studied for trochanteric fractures like intramedullary nailing, the dynamic hip screws, and the use of different plating systems.

Until today, there are only a few studies dealing with trochanteric fractures in children and adolescent patients [1, 17, 20, 21, 23–25] as this is a very rare injury. One of the largest series is the one by Hoekstra et al. [20] investigating 11 children and adolescents with pertrochanteric fractures.

In 1983, Hoekstra et al. [20] presented in their work a series of 11 patients (from 1909 to 1981) of whom 92% were treated conservatively with bedrest alone or in conjunction with a hip spica. Patients had an average hospital stay of 8 weeks and weight bearing started after an average period of 9 weeks. Long-term complications (severe axis deformity—coxa vara—and leg length disturbance) of fracture healing occurred in two patients. Still, all patients were satisfied with the obtained results after a mean follow-up of 18 years. None of the patients had to undergo a further hip surgery during follow-up time [20]. Nowadays, better implants are available which allow immediate full weight bearing (gamma nail) or partial weight bearing (dynamic hip screw, TEN, Prevot nails, and angle plates). The length of hospitalization in our study is much less although 6 out of 20 patients were polytraumatized with a mean hospital stay of 32 days (min. 14; max. 56 days). The other 14 patients had a mean stay of 10 days (min. 3; max. 25 days).

**Fig. 8:**
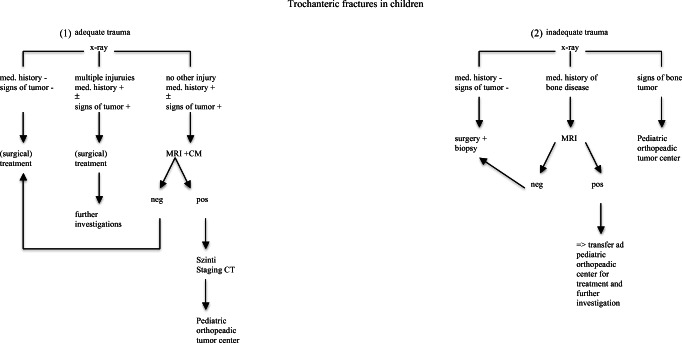
Treatment algorithm. (1) In case of adequate trauma with a negative medical history concerning bone disease and no signs of tumor in the X-ray, surgical treatment is recommended. When multiple injuries are present and also the medical history ± signs of tumor in the X-ray is positive, further investigations are recommended after initial therapy. When no other injury is present but the medical history ± signs of tumor in the X-ray is positive, an MRI plus contrast agent is recommended. When this MRI is negative, treatment can be followed; When MRI is positive, a staging CT and further a transfer to a pediatric orthopedic tumor center should be initiated. (2) In case of inadequate trauma with a negative medical history concerning bone disease and no signs of tumor in the X-ray, surgical treatment and obtaining a biopsy for exclusion of an underlying bone disease are recommended. When the medical history of a bone disease is positive, further imaging should be performed. When bony lesions are negative ≥ surgical treatment; when bony lesions are positive ≥ transfer for further investigation and treatment to a pediatric orthopedic center. When signs of tumor are positive, immediate transfer to a pediatric orthopedic tumor center is recommended

Therefore, we recommend surgical fracture treatment mainly if the fracture is severely displaced, caused by an underlying bone disease (e.g., bone cyst, malignant tumors), or because of additional severe injuries (polytrauma). Therefore, we wanted to provide a treatment algorithm as the following (Fig. 8).

Readers have to be aware of the limitations of this study. This is a retrospective review over a time period of 25 years, including patients diagnosed and treated with different surgical methods. In the last decades, there was a rapid growth in medical imaging techniques and surgical methods. Based on this knowledge, this data analysis has to be reviewed critically. Due to the rarity of this fracture type occurring in children and young adolescent, there were only retrospective studies found in literature. Any prospective study dealing with this rare fracture type would need to be multi-centered. Finally, a meta-analysis could be a good method to provide significant data.

However, this study focused on the presentation of a consecutive single-center experience focusing on the clinical and radiological outcomes after treatment of trochanteric fractures at a level I trauma center.

## Conclusion

Trochanteric femoral fractures in children and adolescents are very rare. Therefore, references regarding treatment modalities are limited. In this collective, an adequate fracture reduction and stabilization yielded excellent long-term results independent from the surgical method.

However, physicians treating pediatric trauma have to be aware of other predisposing diseases when low-energy trauma leads to a trochanteric fracture as in this study, 50% of the trochanteric fractures were associated with bone pathologies.

## Abbreviations

AO: Arbeitsgemeinschaft Osteosynthese
AVN: Avascular necrosis
CT: Computed tomography
MRI: Magnet resonance imaging
